# Facultative cleaning of spiral‐horned antelope by the African paradise flycatcher (*Terpsiphone viridis*)

**DOI:** 10.1002/ece3.9080

**Published:** 2022-07-13

**Authors:** Finote Gijsman

**Affiliations:** ^1^ Department of Ecology and Evolutionary Biology Princeton University Princeton New Jersey USA; ^2^ Mpala Research Centre Nanyuki Kenya

**Keywords:** African savanna, cleaner bird, hawking, Mpala Research Centre, ungulate

## Abstract

In cleaning associations, individuals known as “cleaners” remove and feed on parasites and pests found on, or around, other animals known as “clients.” While best documented in marine environments and as mutualisms, cleaning associations are widespread in terrestrial systems and range along a spectrum of obligate to facultative associations. In African savannas, cleaning associations primarily comprise facultative interactions between mammals and birds that remove attached parasites. Few reports, however, exist on cleaning associations that involve the removal of unattached pests. In this short note, I report a novel facultative bird–ungulate cleaning association involving the removal of unattached pests, between the African paradise flycatcher (*Terpsiphone viridis*) and two species of spiral‐horned antelope (*Tragelaphus* spp.): greater kudu (*Tragelaphus strepsiceros*) and Cape bushbuck (*Tragelaphus sylvaticus*). On multiple occasions, I observed African paradise flycatchers hawking flying insects around greater kudu and a Cape bushbuck during the dry season at the Mpala Research Centre in Laikipia, Kenya. These observations document a rare feeding strategy for the African paradise flycatcher and are among the few records on cleaning interactions involving the removal of unattached pests.

## Graphical abstract

In cleaning associations, individuals known as "cleaners" remove and feed on parasites and pests found on, or around other animals known as "clients". In African savannas, cleaning associations primarily comprise facultative interactions between birds and mammals. Here, I present a novel bird‐ungulate cleaning interaction in an African savanna involving African paradise flycatchers (*Terpsiphone viridis*) and two spiral horned antelope: greater kudu (*Tragelaphus strepsiceros*) and Cape bushbuck (*Tragelaphus sylvaticus*).

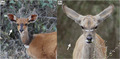

## 1 INTRODUCTION

Cleaning associations, which involve a “cleaner” species that removes and feeds on parasites, debris and other material from a “client” species, are a widespread type of interspecific interaction found in both aquatic and terrestrial ecosystems (Caves, 2021; Sazima et al., 2012). While largely cited as positive interactions, such associations can range in their specificity and/or outcome depending on the species involved, the client's parasite load, type of matter removed by cleaners, and environmental conditions (Caves, 2021; Cheney & Côté, 2005; Vaughan et al., 2017). Cleaning associations can obligate mutualisms if they represent a major food source for cleaners and result in a sizeable reduction in parasites that often inflict painful wounds, act as vectors of pathogens, and/or are a nuisance to clients (Poulin & Grutter, 1996). In other cases, cleaning associations can be facultative, with interactions being largely opportunistic and/or commensal if parasite removal effects are negligible for clients (Caves, 2021; Sazima, 2011). Additionally, the way in which parasites or pests are removed by cleaners can further influence the outcome of these cleaning associations. For instance, clients may benefit from the direct removal of ticks and other attached parasites that they are unable to reach or detach themselves, but they may also benefit from the removal of unattached pests (e.g., insects) that swarm around them or fly into mucous membranes (Palmer et al., 2019).

In terrestrial systems, cleaning associations are dominated by interactions between birds and mammals, a large majority of which are facultative and are reported to occur in Africa (Dean & MacDonald, 1981; Nyaguthii et al., 2021; Sazima, 2011). Cleaner birds in these systems often employ one of two strategies: (1) “gleaning,” which involves the removal of attached parasites by perching on the backs of mammal clients, or (2) “hawking,” which involves the removal of unattached pests by feeding on the wing and returning to a perching site (Dean & MacDonald, 1981; Sazima, 2011). Well‐known examples of African facultative mammal gleaners include red‐ and pale‐winged starlings (Fennessy, 2003; Penzhorn & Horak, 1989), yellow‐bellied bulbuls (Roberts, 1993), African jacanas (Ruggiero, 2008), and others (Dean & MacDonald, 1981; Nyaguthii et al., 2021) that clean ticks and other arthropods. By contrast, few instances of facultative hawkers of insects attracted to wildlife (i.e., unattached pests) exist.

Here, I report a series of novel observations of the African paradise flycatcher (Passeriformes: Monarchidae, *Terpsiphone viridis*) hawking insects around greater kudu (*Tragelaphus strepsiceros*) and Cape bushbuck (*Tragelaphus scriptus*). With the exception of one reported interaction with a red duiker in False Bay Park, South Africa (Dean & MacDonald, 1981), African paradise flycatchers have not previously been documented to forage on insects attracted to wild ungulates. These observations, therefore, document an unusual feeding strategy for the African paradise flycatcher and contribute to a growing body of literature on facultative cleaning associations between birds and ungulates in African savannas.

## 2 METHODS

### 2.1 Species

The African paradise flycatcher is an insectivorous species of passerine bird found across sub‐Saharan Africa, most recognized for its striking appearance. Individuals occur in white and rufous color morphs with males exhibiting long central tail feathers that extend into streamers. To capture insects, African paradise flycatchers employ various feeding strategies, the most common being hawking insects from the air or gleaning them from branches and the undersides of leaves (Branfield, 2012; Fraser, 1983). Tragelaphine antelopes, including Cape bushbuck and greater kudu, are broadly distributed across sub‐Saharan Africa, often in forest or thicket habitats where they browse on woody plants (Khademi, 2017).

### 2.2 Location

**FIGURE 1:**
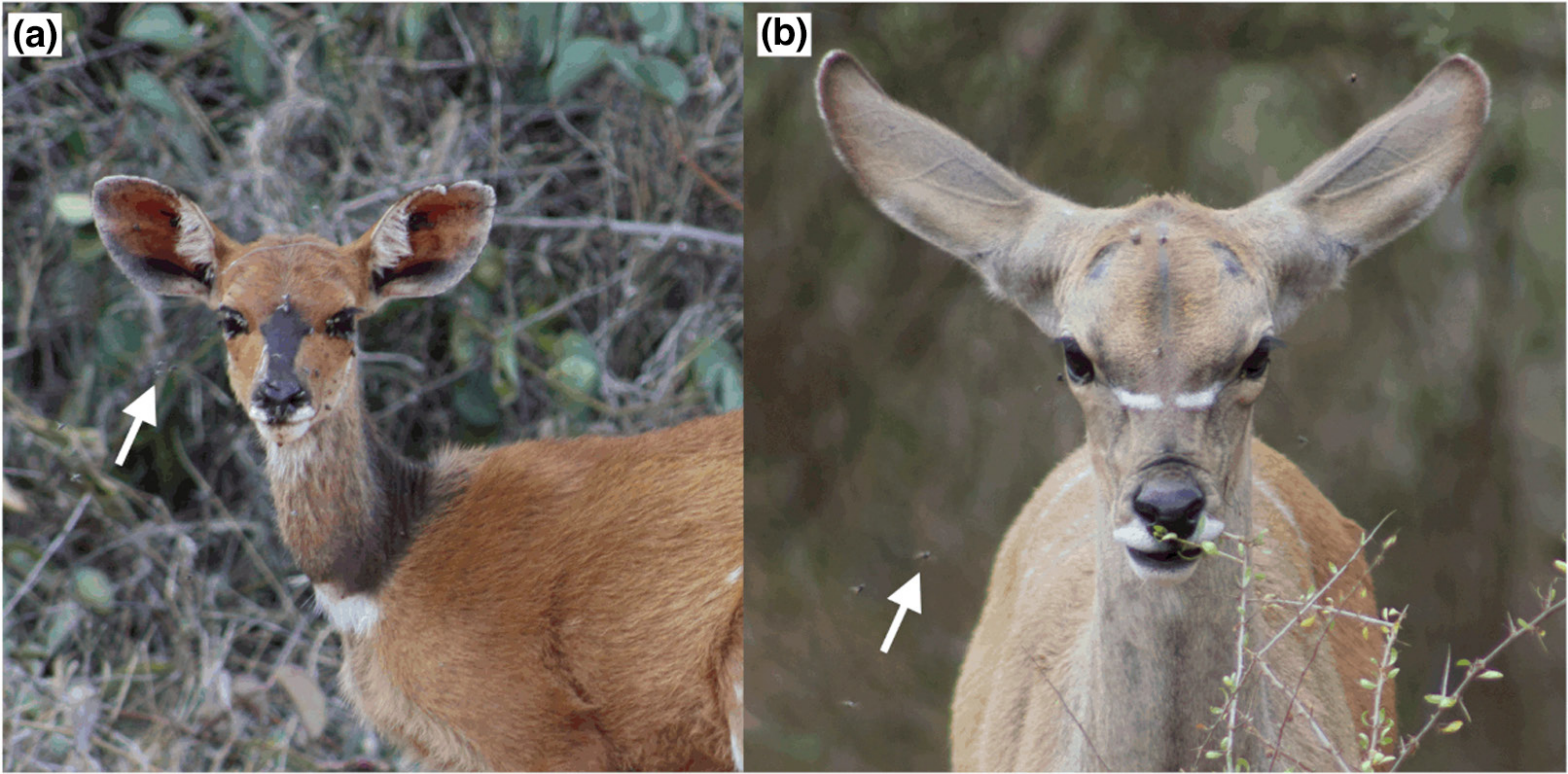
Insects on and flying around (a) Cape bushbuck and (b) greater kudu at the Mpala Research Centre in Laikipia, Kenya

I observed African paradise flycatchers following and hawking unidentified insects around greater kudu and Cape bushbuck at the Mpala Research Centre (MRC, 0°17′ N, 37°52′ E) in Laikipia, Kenya. MRC is a ~ 20,000 ha ranch and wildlife conservancy comprising semi‐arid thorn‐scrub savanna that hosts a great diversity of avian and ungulate wildlife along with domestic cattle, camel, sheep, goat, and donkey (Kartzinel et al., 2019; Young et al., 1997). Both wild and domestic ungulates frequent the field station at the southern end of the property, which is fenced to exclude elephants and accordingly supports dense woody vegetation. Greater kudu typically forage in mixed‐sex groups of 2–5 individuals consisting of adult females, juveniles, and young adult males, while Cape bushbuck forage solitarily. Both species often attract high densities of insects that swarm around them (Figure 1).

## 3 RESULTS

From March 13 to 25, 2022, I observed six cleaning interactions between African paradise flycatchers and greater kudu and one with a lone Cape bushbuck (Table 1, see Video S1). All observed interactions took place during a prolonged dry season in a below‐average rainfall year at MRC (Caylor et al., 2019), between 12 p.m. and 6 p.m.

**TABLE 1:** Cleaning interactions observed between the African paradise flycatcher, greater kudu, and Cape bushbuck in March 2022 at the Mpala Research Centre in Laikipia, Kenya

Ungulate species	Ungulate sex	Interaction date (dd/mm/yy)	Time (hh:mm)	Flycatcher morph	Ungulate group size
Greater kudu	Adult female	13/03/22	15:44	Rufous	2
Greater kudu	Juvenile male	13/03/22	15:58	White	2
Greater kudu	Juvenile male	13/03/22	17:32	White	4
Greater kudu	Adult female	13/03/22	17:33	White	4
Cape bushbuck	Juvenile female	18/03/22	12:47	White	1
Greater kudu	Juvenile female	22/03/22	16:35	Rufous	3
Greater kudu	Adult female	25/03/22	15:19	Rufous	2

**FIGURE 2:**
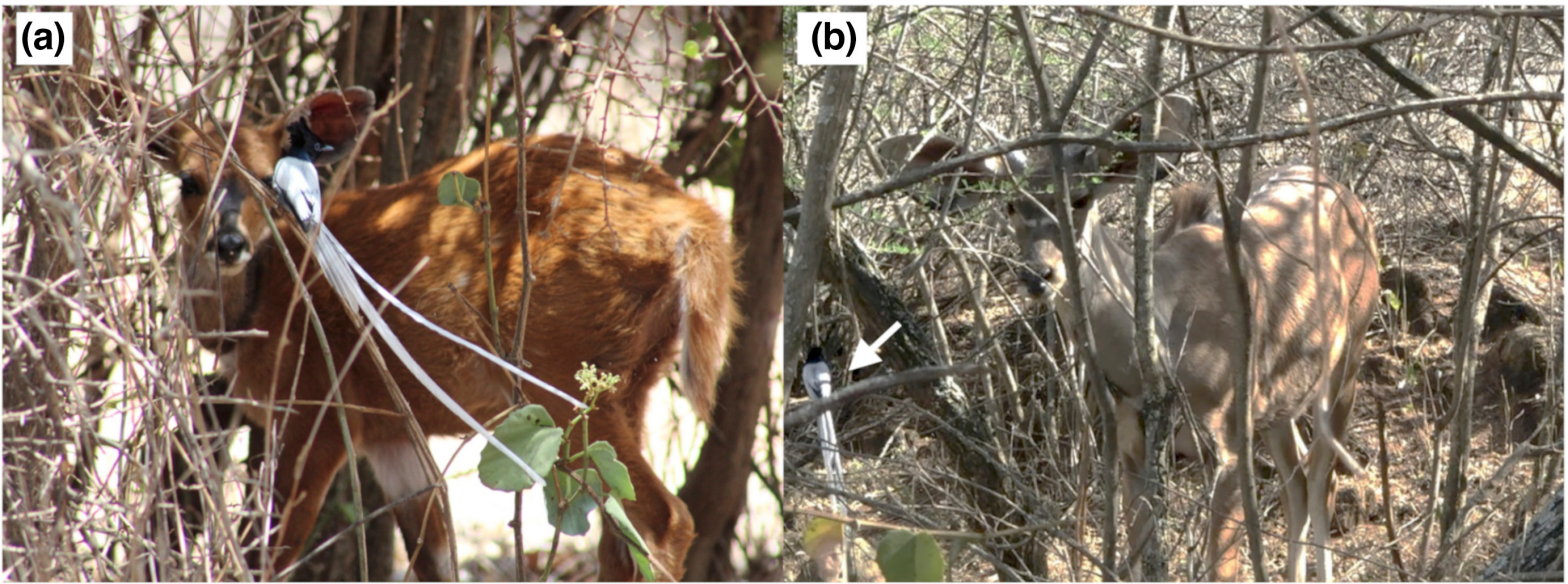
White morph African paradise flycatchers perched near a juvenile (a) Cape bushbuck and (b) greater kudu, waiting to forage on insects flying around them

Throughout each interaction, the greater kudu and Cape bushbuck appeared to remain unbothered and continued to forage as normal, while the flycatchers perched on nearby vegetation and repeatedly performed short pursuit flights around the ungulates' faces and bodies (Figure 2). Both white and rufous flycatcher color morphs were observed foraging on insects around greater kudu, while only the white morph was observed foraging around the Cape bushbuck. The duration of each interaction was determined by the ungulates' foraging patterns with the flycatchers following the greater kudu and Cape bushbuck for as long as they remained within vegetated areas on which they could perch.

## 4 DISCUSSION

While cleaning associations between birds and mammals are a widespread type of interspecific interaction, knowledge on the factors and conditions influencing the establishment of novel and/or persistent interactions between cleaner and client species remains limited. I present what is to my knowledge a previously undescribed cleaning association for the African paradise flycatcher with greater kudu and Cape bushbuck. These observations describe a new bird–ungulate association in African savannas, contribute to the few recorded instances of cleaning associations that involve the removal of unattached pests (but see Palmer et al., 2019, for another example of such an association between bats and white‐tailed deer), and reflect a facultative feeding strategy for the African paradise flycatcher.

Among the multiple feeding strategies that African paradise flycatchers employ to capture invertebrate prey, associating with and hawking insects around wildlife appears to be rare. Only one record on such an association with a red duiker exists (Dean & MacDonald, 1981). The seven cleaning interactions that I observed over the span of 11 days are therefore peculiar and raise the question as to whether such interactions are more common during periods of low insect activity or abundance such as in the dry season and whether such cleaning behaviors have become established in the African paradise flycatcher population at MRC. As no further efforts were made to document these interactions over time, future cross‐seasonal comparisons may provide insight into the broader role that African paradise flycatchers may play as cleaners of African ungulates.

Additionally, it is important to note that these observations took place within the confines of a fenced research center. Numerous reports on cleaning associations between birds and ungulates come from areas of recreation and conservation (D'Angelo et al., 2016; Gijsman & Guevara, 2020), including a recent observation of a black‐cheeked waxbill (*Brunhilda charmosyna*) cleaning a Kirk's dik‐dik (*Madoqua kirkii*), also during the dry season at MRC (Nyaguthii et al., 2021). Two hypotheses could potentially explain this pattern. First, recreation and conservation areas may provide researchers with more opportunities to interact with wildlife and thus easily record associations that would otherwise be hard to observe in more remote natural areas (Lopez et al., 2020). Second, recreation or human‐frequented areas may also indirectly promote novel species interactions by attracting species seeking anthropogenic food subsidies (Birnie‐Gauvin et al., 2017; Marzluff, 2001) or spatial refugia from predators (Leighton et al., 2010; Muhly et al., 2011; Suraci et al., 2019). For instance, greater kudu and Cape bushbuck regularly visit MRC to forage within its protective enclosure and often do so with remarkably predictable foraging routes and patterns (FG, *pers. obs*.). Such routine foraging patterns may thus facilitate cleaning interactions with African paradise flycatchers by reinforcing learning processes via repeated encounters with parasite‐ or pest‐laden ungulates (Dean & MacDonald, 1981).

Lastly, as greater kudu and Cape bushbuck often attract large swarms of insects and are important blood meal sources for biting insects like tsetse flies across much of their ranges (Gaithuma et al., 2020; Moloo, 1993), such associations can be beneficial if they substantially reduce the prevalence and abundance of disease‐carrying insects flying around them. Analogously, by associating with ungulates swarmed by insects, African paradise flycatchers may benefit from a reduction in prey search time and increase their likelihood of finding food. Whether the benefits accrued by both partners through these interactions are considerable, however, is unknown and would depend on the number and types of insects that were removed by flycatchers from the airspace around the ungulates – both of which I was unable to ascertain through these observations. Further investigations may elucidate whether these types of associations are frequent occurrences and a common feeding strategy for African paradise flycatchers and other cleaning birds.

## AUTHOR CONTRIBUTIONS

**Finote Gijsman:** Conceptualization (lead); data curation (lead); funding acquisition (lead); investigation (lead); writing – original draft (lead); writing – review and editing (lead).

## CONFLICT OF INTEREST

The author also declares no conflict of interest.

## Supporting information


Video S1

## Data Availability

Data collected for this project are available in Table 1 of this manuscript.
